# Development of Poly(2-Methacryloyloxyethyl Phosphorylcholine)-Functionalized Hydrogels for Reducing Protein and Bacterial Adsorption

**DOI:** 10.3390/ma13040943

**Published:** 2020-02-20

**Authors:** Temmy Pegarro Vales, Jun-Pil Jee, Won Young Lee, Sung Cho, Gye Myung Lee, Ho-Joong Kim, Jung Suk Kim

**Affiliations:** 1 Department of Chemistry, Chosun University, Gwangju 501-759, Korea; valestemmy@gmail.com (T.P.V.); hjkim@chosun.ac.kr (H.-J.K.); 2 Department of Natural Sciences, Caraga State University, Butuan City 8600, Philippines; 3 College of Pharmacy, Chosun University, Gwangju 501-759, Korea; jee@chosun.ac.kr (J.-P.J.); ovilion777@naver.com (W.Y.L.); 4 Department of Chemistry, Chonnam National University, Gwangju 61186, Korea; scho@chonnam.ac.kr; 5 Department of Carbon Materials, Chosun University, Gwangju 61452, Korea; vkehdi@korea.kr; 6 Department of Orthopaedic Surgery, Graduate School, College of Medicine, Kyung Hee University, Seoul 02447, Korea

**Keywords:** biomembrane-mimicking, hydrogel, protein adsorption, surface-functionalization, wettability

## Abstract

A series of hydrogels with intrinsic antifouling properties was prepared via surface-functionalization of poly(2-hydroxyethyl methacrylate) [p(HEMA)]-based hydrogels with the biomembrane-mimicking zwitterionic polymer, poly(2-methacryloyloxyethyl phosphorylcholine) [p(MPC)]. The p(MPC)-modified hydrogels have enhanced surface wettability, high water content retention (61.0%–68.3%), and good transmittance (>90%). Notably, the presence of zwitterionic MPC moieties at the hydrogel surfaces lowered the adsorption of proteins such as lysozyme and bovine serum albumin (BSA) by 73%–74% and 59%–66%, respectively, and reduced bacterial adsorption by approximately 10%–73% relative to the unmodified control. The anti-biofouling properties of the p(MPC)-functionalized hydrogels are largely attributed to the dense hydration layer formed at the hydrogel surfaces by the zwitterionic moieties. Overall, the results demonstrate that biocompatible and antifouling hydrogels based on p(HEMA)-p(MPC) structures have promising potential for application in biomedical materials.

## 1. Introduction

Significant interest has emerged in the development and design of functional biomaterials with excellent biocompatibility and antibiofouling properties. A promising biomaterial that has long been investigated for diverse biomedical and industrial applications is the hydrogel, which consists of three-dimensional cross-linked hydrophilic polymeric networks capable of retaining large amounts of water and biological fluids [1,2]. Parhi has comprehensively reviewed the different types of cross-linking-based hydrogels for various pharmaceutical applications including small-molecule cross-linked hydrogels, polymer-polymer cross-linked hydrogels, photo cross-linked hydrogels, enzymatic cross-linked hydrogels, and interpenetrating networks [3]. In particular, poly(2-hydroxyethyl methacrylate) [p(HEMA)]-based hydrogels have been successfully commercialized worldwide as bulk materials for the development and production of biomedical materials such as urethral catheters [4], neural tissue devices [5], dental adhesives [6], implantable drug delivery systems [7], and contact and intraocular lenses [8,9,10,11]. This is largely due to their essential and desirable attributes, including high water retention capacity, broad biocompatibility, and physical features that resemble living soft tissues [12]. Moreover, p(HEMA)-based hydrogels are more inert to normal biological processes, permeable to metabolites, and are capable of withstanding a very high temperature sterilization without incurring physical damage in comparison to other types of hydrogels such as poly(ethylene glycol) diacrylate (PEGDA), gelatin methacryloyl (GELMA), and others [13]. While p(HEMA) hydrogels exhibit excellent biocompatibility, its fouling, albeit low, is still higher than other nonfouling materials. Moreover, its hydration is lower in comparison to that of native tissue [14], which is actually a particular major issue impeding the development of functional hydrogels: the adherence of proteins and other foulants following the implantation of these biomaterials in the biological environment [15]. Nonspecific adsorption of protein and other biomolecules plays a significant role in the development of undesirable biological responses, including inflammation, blood clotting, biofilm formation, cell adhesion, bacterial adhesion, and bacterial infection [16]. Thus, there is a growing need to develop biomaterials that inhibit or reduce nonspecific protein adsorption and resist bacterial adhesion and colonization.

The surface functionalization utilizing second hydrophilic polymers such as polysaccharides (e.g., dextran and hyaluronic acid and) [17,18], poly(ethylene glycol) (PEG) [19,20,21,22], and biomembrane-mimicking zwitterionic polymers [e.g., 2-methacryloyloxyethyl phosphorylcholine (MPC) and carboxybetaine methacrylate (CBMA)] [23,24,25] is one such strategy to develop such “protein-resistant” biomaterials. These polymers are incorporated on biomaterial surfaces using a diverse set of surface coating and modification techniques, including physical adsorption, graft polymerization, self-assembled monolayers (SAMs), layer-by-layer assembly, interpenetrating polymer network (IPN), surface-initiated atom transfer radical polymerization (ATRP), and conventional free-radical polymerization [26,27,28,29,30,31,32,33,34]. Among these methods, the free-radical polymerization seems the most common reaction employed in the development of surface-modified hydrogels. Recently, a review article by Choi et al. highlighted the recent advances on the most commonly used photocross-linkable hydrogels, including PEGDA, GELMA, and methacrylated hyaluronic acid for several biomedical applications [35].

One of the recognized next-generation antifouling biomaterials is the zwitterionic polymers, largely due to their ability to form hydration shell via electrostatic interactions, bringing about tighter and denser adsorbed water [36,37]. There are two main classes comprising the zwitterionic polymers namely polybetaines and polyampholytes. The typical polybetaines such as poly(sulfobetaine methacrylate) [p(SBMA)], poly(carboxybetaine methacrylate) [p(CBMA)], and poly(2-methacryloyloxyethyl phosphorylcholine) [p(MPC)] have their charged groups located on the pendant side chains of same monomer units [38]. Polyampholytes, on the other hand, are zwitterionic polymers whose charged groups are located on different monomer units and can be prepared from copolymerization of equimolar quantities of positively charged and negatively charged monomers. Schroeder, et. al. have prepared multifunctional polyampholytes hydrogels comprising positively charged [2-(acryloyloxy)ethyl] trimethylammonium chloride (TMA) and negatively charged 2-carboxyethyl acrylate (CAA) moieties [39]. However, one difficulty usually encountered with using polyampholytes as antifouling materials is that the charged groups are not homogeneously arranged. It is imperative to have surfaces with charged groups that are homogeneously arranged to minimize the effects of any acid−base interactions with the material and achieve an optimal environment in generating a densely hydration layer [40].

Among a wide range of biocompatible zwitterionic polybetaines, poly(2-methacryloyloxyethyl phosphorylcholine) [p(MPC)] appears to be a promising anti-fouling biomaterial. MPC-containing polymeric materials are used as anti-fouling agents because the external surface of cellular membranes is rich in phospholipids possessing zwitterion headgroups, particularly phosphatidylcholine [41]. Hence, MPC-surface-modified biomaterials are likely to mimic cell membranes and be highly biocompatible [42]. Several biocompatible polymers such as polyethylene, segmented polyetherurethane (SPU), siloxane polymers, and poly(tetrafluoroethylene) (PTFE) have been modified to generate surfaces with antifouling properties [43,44,45]. The anti-fouling properties of these MPC-coated surfaces are highly correlated with the formation of an excellent hydration layer. This hydration layer leads to a strong repulsive force against the surrounding proteins and prevents the generation of any prime layer that might otherwise surfaces are strongly related to the large fraction of free water retained around the polymeric chains, which facilitate microorganisms to anchor onto the surface [46,47].

Herein, we prepared a series of p(MPC)-surface-functionalized [p(HEMA)] hydrogels employing a facile free-radical polymerization reaction, and the adsorption of protein and bacteria onto the p(MPC)-coated surfaces was evaluated. Comprehensively studied bovine serum albumin (BSA) and lysozyme were selected as model proteins [48], while gram-negative Escherichia coli was used for the in-vitro bacterial desorption experiment. Furthermore, the effects of surface-functionalization with p(PMC) on surface wettability, equilibrium swelling, and optical transmittance of the resultant hydrogels were investigated.

## 2. Materials and Methods

### 2.1. Chemicals and Equipment

Hydroxyethyl methacrylate (HEMA), ethylene glycol dimethacrylate (EGDMA), lysozyme (chicken egg lysozyme), and BSA were obtained from Sigma Aldrich (St Louis, MO, USA). 2-Methacryloyloxyethyl phosphorylcholine (MPC) was obtained from KCI Limited (Seoul, Korea). DifcoTM Mueller-Hinton broth medium was purchased from Thermo Fisher Scientific (Leicestershire, UK), while azobisisobutyronitrile (AIBN) and benzophenone were procured from Junsei (Tokyo, Japan) and Daejung Chemical & Metal Co. (Shiheung, Korea), respectively. Acetone, trifluoroacetic acid, and other solvents were of analytical grade and used without further purification. The transmittance spectra of the hydrogels were measured at 300–700 nm, in triplicate, using a Shimadzu ultraviolet–visible spectrophotometer 1601 PC (UV-1650PC) (Tokyo, Japan).

### 2.2. Synthesis of HEMA-Based Hydrogels

The HEMA monomer was initially vacuum-distilled before polymerization. Subsequently, EGDMA (0.04 g) and AIBN (0.04 g) were dissolved in HEMA (9.92 g) and mixed for 30 min. The mixture was then injected into a square mold comprising two glass plates internally covered with a polypropylene sheet and separated by a 0.20 mm-width Teflon frame. The samples were heated at 90 °C for 5 h to allow the polymerization to take place. After cooling to room temperature, samples were removed from the molds and placed in 400 mL de-ionized water for extensive dialysis. To completely remove unreacted monomer and initiators, the dialysis was carried out for 2 d by changing the water three times per day. Afterwards, square hydrogels (10 mm × 10 mm × 2.4 mm) were cleaved from the sample, submerged in boiling water for 15 min, and dried at 40 °C overnight.

### 2.3. Surface-Functionalization of p(HEMA) Hydrogels with p(MPC)

The p(HEMA) hydrogels were surface-functionalized with p(MPC) according to a previously reported procedure [34]. All reactions were carried out at room temperature. Initially, the p(HEMA) substrates were initially submerged in a benzophenone solution in acetone (10 mg/mL) for 1 min and vacuum-dried for 1 h. Subsequently, p(HEMA) hydrogels with adsorbed benzophenone were immersed in a series of aqueous MPC solutions with monomer concentrations of 0.1 M, 0.25 M, and 0.5 M. The samples were irradiated with UV-B (~320 nm) using a customized lamp chamber composed of eight 15W G15T8E lamps (Sankyo-Denki, Tokyo, Japan). After 15 min, the samples were removed from the lamp chamber and thoroughly washed with acetone and water to remove unreacted monomers, excess benzophenone, and benzopinacol by-products. To further eliminate any non-covalently attached polymer and excess acetone, MPC-surface-functionalized hydrogels were soaked in deionized water overnight.

### 2.4. Optical Transmittance Measurement of the p(HEMA)/p(MPC) Hydrogels

The transmission spectra of the hydrogels (average thickness of the prepared hydrogels = 2.4 mm) were obtained using a Shimadzu, UV-1650PC spectrophotometer (Tokyo, Japan). Each measurement was carried out at a wavelength range of 300–700 nm at room temperature, with each measurement performed four times; here, the averaged results are reported.

### 2.5. Equilibrium Swelling of the p(HEMA)/p(MPC) Hydrogels

The equilibrium swelling ratio (ESR) of the prepared hydrogels were measured gravimetrically at room temperature. Initially, all hydrogels were fully dried to a constant weight before the equilibrium swelling. Subsequently, the fully dehydrated hydrogels were allowed to swell to equilibrium by immersion in phosphate-buffered saline (PBS, pH 7.4) and kept at room temperature for 24 h. It is widely known that a 24-h equilibration time is sufficient for fully swelling hydrogel samples [49,50]. Then, samples were removed from the PBS, excess surface water was blotted off with tissue paper, and the weights of the swollen hydrogels were recorded. The ESR was calculated according to the following equation:

ESR (%) = [(W_s_ − W_d_)/W_d_] × 100 (1)

where W_s_ and W_d_ denote the hydrogel weights at the equilibrium swelling and dry states, respectively. The average values of three measurements are reported for each sample.

### 2.6. Contact Angle Measurement of the p(HEMA)/p(MPC) Hydrogels

The surface wettability of the hydrogels was characterized by the water contact angle. Each sample was measured using a drop shape analyzer (DSA100, Krüss GmbH, Hamburg, Germany). A drop (4.5 μL) of nanopure water was positioned on the hydrogel surface and the static water contact angle was determined from the image using imaging software. Ten measurements were performed, and the averaged results are reported.

### 2.7. Protein Desorption from the p(HEMA)/p(MPC) Hydrogels

Protein desorption was measured previously described [51]. The prepared hydrogels were initially immersed in 10 mL PBS (pH 7.4) and incubated in an incubator shaker at room temperature and 150 rpm for 24 h. Two artificial tear solutions containing 3.88 g/L of bovine serum albumin (BSA) and 1.20 g/L of chicken egg-white lysozyme in PBS were prepared. Subsequently, five samples of each hydrogel type were placed in a vial containing 10 mL of artificial tear solution and incubated at 37 °C and 150 rpm for 12 h. Afterwards, the hydrogels were rinsed with PBS after the first period of incubation to remove unbound protein on the hydrogel surface, then rapidly placed into 0.1% trifluoroacetic acid in an acetonitrile/water 1:1 (v/v) extraction solvent and incubated in the dark at room temperature for 24 h. The extracted solution was diluted to 1/10th using the mobile phase and then subsequently analyzed. A reverse-phase high-performance liquid chromatography system (HPLC Plus, Azura, Germany) was employed to quantify the concentration of proteins extracted from the hydrogels. Approximately 20 µL of the mixed solution was injected into a C18 column (LUNA-C18, 4.6 mm × 250 mm, 5 μm; Phenomenex, Torrance, CA, USA), and eluted using an isocratic mobile phase that was a mixture of 50% acetonitrile containing 0.1% trifluoroacetic acid and 50% water. The run time and flow rate were set to 20 min and 1.0 mL/min, respectively, and the samples were detected at 220 nm using a UV-detector (DAD 2.1L, Azura, Germany).

### 2.8. In Vitro Bacterial Desorption Test of the p(HEMA)/p(MPC) Hydrogels

All hydrogel samples were sealed in a 70% ethanol solution and transferred to sterile distilled water 1 day before the bacterial desorption experiment. A Mueller-Hinton medium solution was prepared to culture E. coli and approximately 1 mL of an E. coli stock solution was added to 50 mL of 21 g/L sterilized Mueller-Hinton medium solution and incubated at 37 °C for 6 h. Subsequently, approximately 2 mL of the cultured E. coli solution was inoculated in 400 mL of sterilized Mueller-Hinton medium solution and incubated with mild shaking at 37 °C for 12 h. After the incubation, the resultant E. coli solution was transferred into a 1L beaker, and three samples of each type were placed into a stainless-steel net-shaped container and immersed in the bacterial solution. The samples were placed in an orbital shaker at room temperature, and the bacteria were allowed to reach the sample surfaces evenly. After 6 h of incubation, the samples were quickly washed with sterile distilled water and incubated in 20 mL of sterilized Mueller-Hinton medium in an incubator shaker set at 37 °C and 150 rpm. At a pre-determined time, approximately 1 mL of the culture samples was taken and absorbance was measured at 595 nm using a UV spectrophotometer (TU-1800, Korea Instruments Co., Ltd., Seoul, Korea).

### 2.9. Statistical Analysis

Student’s t-test was used to statistically analyze unpaired data and compare the two mean values. In comparing more than two mean values, one-way analysis of variance (ANOVA) followed by Tukey’s multiple-comparison test was employed. The p-value < 0.05 was considered statistically significant in all analyzes. All data were expressed as mean ± standard deviation.

## 3. Results and Discussion

**Scheme 1:**
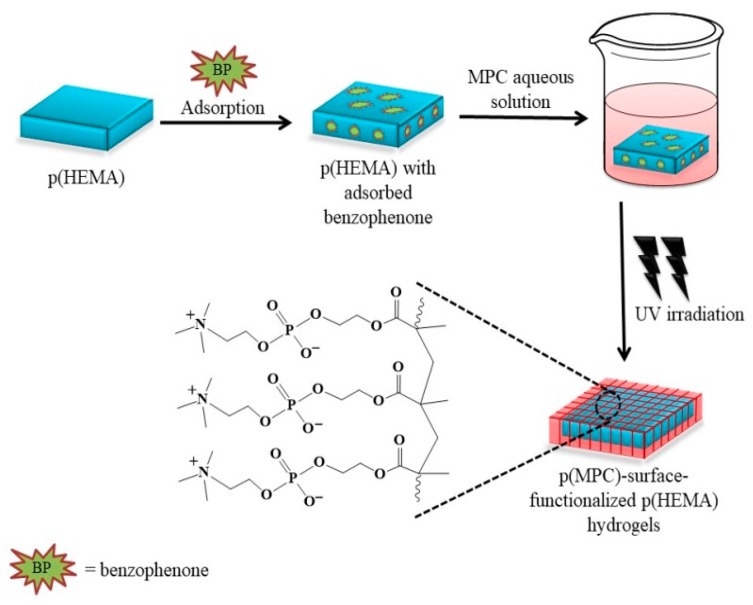
Schematic illustration for the development of poly(2-hydroxyethyl methacrylate) [p(HEMA)]-based hydrogels surface-functionalized with biomimetic poly(2-methacryloyloxyethyl phosphorylcholine) [p(MPC)].

A series of antifouling hydrogels was prepared by following the three-step synthesis pathway presented in Scheme 1. Initially, the HEMA monomers were polymerized using EGDMA and AIBN as the cross-linking agent and free radical source, respectively. Upon thermal activation (~90 °C), the AIBN decomposes to two 2-cyanopropyl fragments with unpaired electrons that rapidly react with the nearby HEMA monomers and EGDMA crosslinkers hereby producing growing HEMA polymer chains crosslinked with EGDMA moieties. The subsequent two steps involved adsorption of benzophenone as a photosensitizer that exhibits a well-established photochemical reaction and the UV-induced free radical polymerization of MPC monomers onto the hydrogel surfaces. The irradiation around ~320 nm converts the benzophenone into its excited triplet state, which abstracts a hydrogen atom from the α-methyl group of the nearby MPC units to generate both the benzophenone radicals and MPC radicals capable of initiating polymerization of the MPC monomers onto the hydrogel surfaces [52]. Surface-functionalization was conducted in an aqueous environment with MPC concentrations of 0.10 M, 0.25 M, and 0.50 M, each solution generating a hydrogel with intrinsic antifouling properties: p(HEMA)/p(MPC)0.1, p(HEMA)/p(MPC)0.25, and p(HEMA)/p(MPC)0.5.

As listed in Table 1, the hydrogels produced by a higher concentration of MPC showed higher amounts of p(MPC) on the substrate surfaces when compared to those produced by lower MPC concentrations. This result was highly anticipated because when there are more MPC monomers available for the polymerization reaction, the coverage of the hydrogel surfaces by the hydrophilic MPC moieties is increased. The quantities of p(MPC) per hydrogel surface of p(HEMA)/p(MPC)0.5, p(HEMA)/p(MPC)0.25, and p(HEMA)/p(MPC)0.1 were estimated at ~24.0, ~21.8, and ~18.0 μmol/cm^2^, respectively. The amounts of the conjugated p(MPC) in the synthesized hydrogels were determined using gravimetric measurements. Moreover, the effect of surface-functionalization with poly(MPC) on the hydrogel equilibrium swelling properties was investigated in a PBS medium at room temperature. High equilibrium water content is one of the typical features associated with hydrogels and various factors are known to control and affect the hydrogel swelling behavior, including the degree of ionization and the resulting interaction with counter ions, hydrophilic/hydrophobic balance of the hydrogels, and the hydrogel’s effective crosslinking density [53]. Furthermore, the equilibrium swelling ratio (ESR) value increased, although not significantly, after the surface modification of the p(HEMA) hydrogels with p(MPC). The ESR of pristine p(HEMA) hydrogels was estimated at 60.1%, while that of p(MPC)-modified hydrogels ranged from 61.0% to 68.3% (p > 0.05). It should be noted that the hydrogel’s swelling behavior was largely influenced by the effective crosslinking density of the p(HEMA) network as manifested by the negligible effect of the surface modification on the ESRs of the prepared hydrogels. Nevertheless, the obtained ESRs of hydrogels were in the range of low and intermediate water content (60–83%), which reflect the optimal conditions for natural cartilage and high water content contact lenses [54,55].

**Figure 1:**
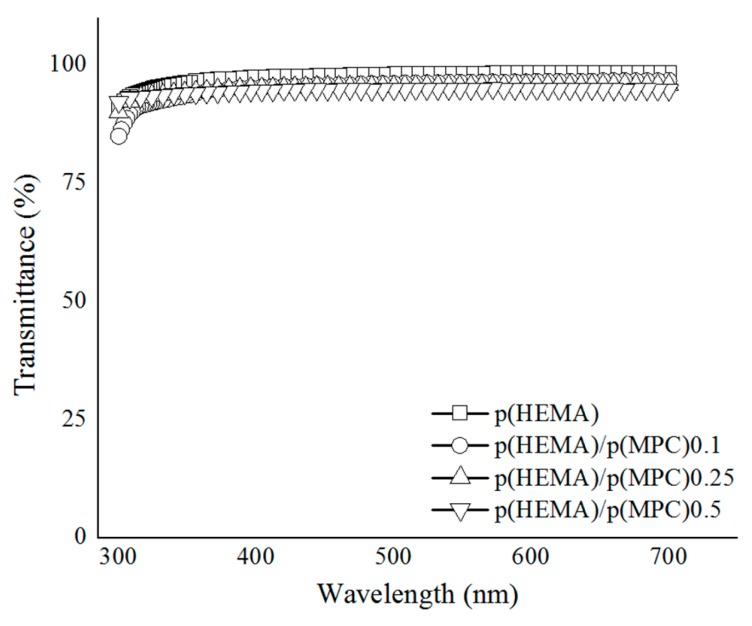
Transmittance spectra of the poly(2-methacryloyloxyethyl phosphorylcholine)-surface-functionalized hydrogels.

Another key and integral attribute that hydrogels require as a material for colorimetric sensors and ophthalmologic materials such as contact lenses is optical transparency. Here, the optical transmittance of the prepared hydrogels was measured at 300–700 nm and reported as % transmittance. As shown in Figure 1, all the hydrogels have high transmittance values (> 94%) that exceed the light transmission required by wearable contact lenses (92%) [12]. Although a decrease of the transmittance of the hydrogels around 300-320 nm range was observed which could be due to the presence of the residual photoinitiator, such transmittance decrement is quite typical around the said wavelengths. Li, et al. measured the transmittance of a commercial contact lens, ACUVUE^®^, and found that the transmittance suddenly decreased below 350 nm due to the UV protection treatment. Typically, benzophenone or benzotriazole is incorporated to absorb the UV light [56]. Nevertheless, the prepared MPC-functionalized hydrogels was considered as being optically transparent, demonstrating the suitability of the prepared hydrogels as contact biomaterials in terms of light transmittance in the range of visible light wavelengths. It is widely recognized that the immiscibility of the monomers largely governs the overall optical transmittance of the hydrogels. Notably, the high % transmittance of the hydrogels indicate that surface-functionalization of the p(HEMA) hydrogels with p(MPC) did not result in opaque or phase-separated hydrogels, which is attributed to the excellent miscibility of the MPC with the monomer solutions containing HEMA and EGDMA.

**Figure 2:**
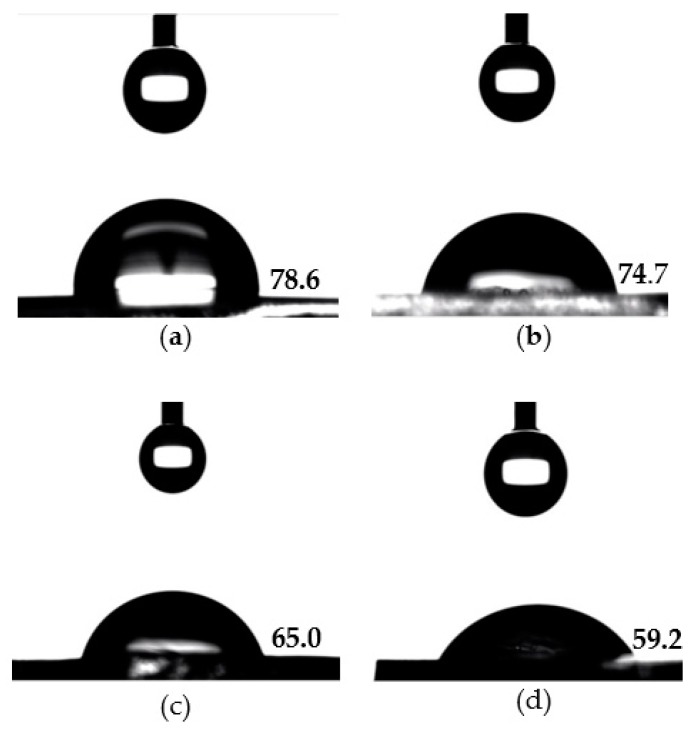
Contact angles of nanopure water droplets (4.5 μL) on (**a**) pristine p(HEMA) hydrogel; (**b**) p(HEMA)/p(MPC)0.1 hydrogel; (**c**) p(HEMA)/p(MPC)0.25 hydrogel; and (**d**) (HEMA)/p(MPC)0.5 hydrogel.

The surface wettability of the prepared hydrogels was evaluated by carrying out contact angle measurements at the liquid-solid interface. The water contact angle measurements of the hydrogels are shown in Table 1 and Figure 2. The surface-functionalization of p(HEMA) hydrogels with zwitterionic and hydrophilic MPC moieties clearly reduced the contact angles and enhanced the surface wettability. A relatively high-water contact angle of 78.6° was observed on the surface of the pristine p(HEMA) hydrogels. Subsequently, the surfaces of p(HEMA)/p(MPC)0.1, p(HEMA)/p(MPC)0.25, and p(HEMA)/p(MPC)0.5, had contact angles of 74.7°, 65.0°, and 59.2°, respectively. Furthermore, the statistical analysis of the obtained water contact angles indicated that a significant difference (p < 0.05) was observed only after the surface-functionalization of the p(HEMA) substrate with 0.25 M MPC, signifying the crucial role of the zwitterionic MPC played in increasing the wettability of the hydrogel surface. The increased surface wettability of the prepared hydrogels is attributed to the ionic hydration of the phosphorylcholine group in the MPC unit. Such increased surface wettability has also been reported for MPC-surface-functionalized biomaterials [57,58,59].

In addition, only p(HEMA)/p(MPC)0.5 among all MPC-modified hydrogels showed significant difference on its water contact angle after being compared to p(HEMA)/p(MPC)0.1, signifying that the best hydration layer was achieved after employing 0.50 M MPC. It is quite expected that higher MPC concentrations would induce higher wettability than that of the hydrogels modified at lower MPC concentrations, since denser p(MPC) hydration layers are bound to the surfaces of the hydrogels prepared at high MPC concentrations.

**Figure 3:**
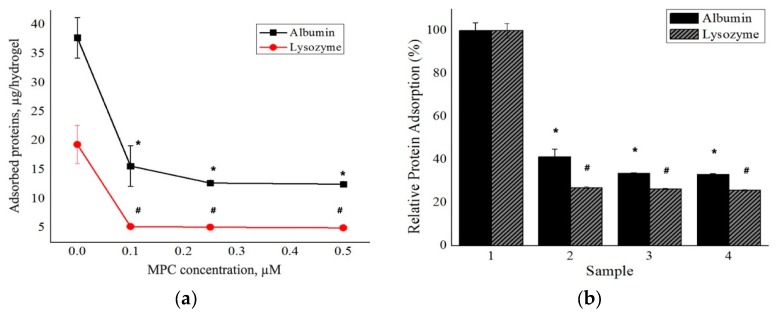
Protein adsorption profiles of the poly(2-methacryloyloxyethyl phosphorylcholine)-surface-functionalized hydrogels in terms of (**a**) mass of adsorbed proteins per hydrogel and (**b**) relative protein adsorption (%) (*,# = p < 0.05 compared to the control). Legend: 1 = p(HEMA)-H; 2 = p(HEMA)/p(MPC)0.1; 3 = p(HEMA)/p(MPC)0.25; 4 = p(HEMA)/p(MPC)0.5.

Another important goal in the preparation of biomaterials is to create surfaces with non-fouling properties, to prevent any serious and unwanted biological responses and reactions [60]. Protein deposition onto biological surfaces is affected by several factors including surface chemistry, topology, protein structure, size, and charge [61,62]. Here, we evaluated the adsorption of two model proteins, BSA and lysozyme, to the prepared hydrogels. As shown in Figure 3, the amounts of BSA adsorbed onto the surfaces of p(HEMA)/p(MPC)0.1, p(HEMA)/p(MPC)0.25, and p(HEMA)/p(MPC)0.5 hydrogels were estimated to be 15.7, 12.7, and 12.5 µg/hydrogel, respectively. Compared to the 37.8 µg/hydrogel observed for the unmodified hydrogels, this result corresponds to reduced BSA adsorption by approximately 59%, 66%, and 67%, respectively.

Moreover, MPC-surface-functionalization also decreased lysozyme adsorption on the hydrogels. The unmodified p(HEMA) hydrogels adsorbed approximately 19.4 µg/hydrogel of lysozyme onto their surfaces while p(MPC)-modified surfaces adsorbed 5.0–5.2 µg/hydrogel, a roughly 73–74% reduction in lysozyme adsorption. Moreover, a significant difference (p < 0.05) was observed for both BSA and lysozyme adsorption profiles of all the MPC-functionalized hydrogels when compared to the pristine p(HEMA) hydrogels. This signifies that the presence of p(MPC) have contributed to the suppression of BSA and lysozyme adsorption onto the hydrogel surfaces. Furthermore, no significant difference was observed when the mean values of all MPC-functionalized hydrogels were compared to one another. Overall, the obtained protein adsorption profiles of the prepared hydrogels demonstrate that the presence of zwitterionic p(MPC) layers on the surfaces of the p(HEMA) hydrogels effectively prevents the adsorption of both BSA and lysozyme. The resistance to protein adsorption is attributed to the interactions between the phosphorylcholine moieties and the surrounding water. A substantial quantity of free water effectively forms a dense hydrated layer around the phosphorylcholine group and acts as a “barrier” that repels proteins and is even thought to inhibit the consequent conformational changes of the adsorbed proteins [63,64,65]. It has been well established that the reduction of protein adsorption by the p(MPC) is highly correlated with the water interactions at the polymer surface. Ishihara et al. [66], one of the leading groups investigating the reduction of protein adsorption by MPC-based polymers, observed that the p(MPC) chains formed unique type of hydration layers when compared to other types of hydrophilic polymers such as polyacrylamide [p(AAm)], poly(ethylene oxide) (PEG), and poly(N-vinyl pyrrolidone) [p(VPy)]. The hydration of p(MPC) mainly arises through hydrophobic hydration of the three methyl groups in the trimethylammonium moiety, inducing an increase in a clathrate cage structure of nearby water molecules and forming an ice-like water state. Hence, MPC-modified surfaces cannot interact strongly with the nearby proteins and cells. Moreover, Ishihara et al. [47] determined the amount of free water on their surfaces using differential scanning calorimetric measurements and observed that these polymers have huge fraction of free water on their surfaces when compared to that of without MPC. In addition, they investigated the change on the conformations of the adsorbed proteins employing ultraviolet and circular dichroism (CD) spectroscopic measurements. The CD spectra revealed that the secondary structure of BSA did not change even after being adsorbed onto the surfaces of MPC-based polymers. The α-helix content was also found to be the same level as in the native state, while it dropped by contact with p(HEMA) substrate. These observations signify that the very high level of free water on the polymer surface provides stabilization to proteins and cells, when these foulants are adsorbed on the polymer surface. Hence, the free water fraction must be one of the more important factors to consider in the protein adsorption resistivity and the subsequent nonbiofouling property of the biomedical polymers. Other groups also reported the excellent performance of the p(MPC)-functionalized surfaces in the reduction of protein adsorption and highly correlated it with the dense hydration layer formed at the hydrogel surfaces [67,68,69].

**Figure 4:**
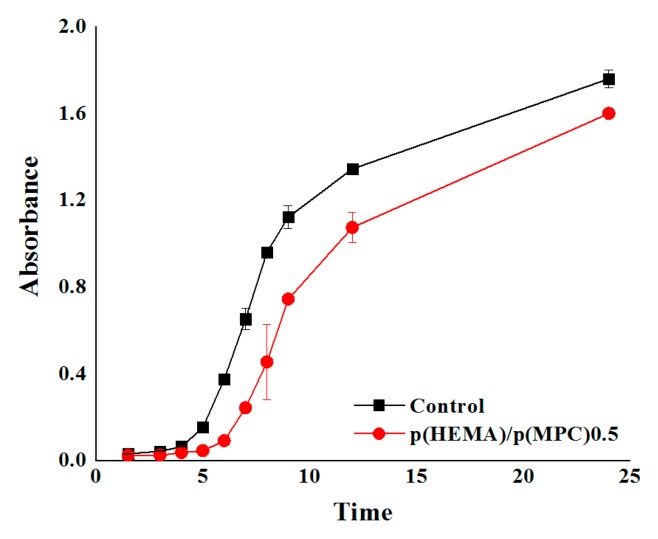
Bacterial adsorption profiles of the poly(2-methacryloyloxyethyl phosphorylcholine)-surface-functionalized hydrogels.

Bacterial adsorption to the prepared hydrogels was investigated using a gram-negative *E. coli*. The p(HEMA)/p(MPC)0.5 hydrogel was assessed as the optimum surface-functionalized hydrogel in this series and selected for the bacterial adsorption experiment, after taking into account the results from the protein adsorption experiments. The best hydration layer and better equilibrium swelling were also observed in p(HEMA)/p(MPC)0.5 hydrogels. The hydrogels were initially immersed in a bacterial suspension of known concentration, incubated for a certain amount of time at 37 °C, removed from the bacterial suspension and re-immersed in a sterile nutrient medium. Then, the bacterial concentration was evaluated at pre-determined time intervals from 1 to 24 h [70] by measuring the optical density of the bacterial suspensions using a UV-spectrophotometer. As shown in Figure 4, there is a significant distinction between the adherent bacterial colonies of the two samples. The suspensions containing the p(MPC)-surface-functionalized hydrogels have lower absorbance values than those of the unmodified hydrogels throughout the 24-h incubation time. These lower absorbance values of the bacterial suspensions correspond to bacterial adsorption values that are reduced by approximately 10–73% of the values of pristine p(HEMA) hydrogels throughout the 24-h incubation period.

Similar to the explanation of protein desorption from prepared hydrogels, the observed reduction in bacterial adsorption onto the hydrogel surfaces may be attributed to the dense hydration layer formed by the zwitterionic p(MPC) on the hydrogel surface [35,36]. This hydration layer leads to a strong repulsive force against surrounding microorganisms, which prevents them from gaining anchorage and subsequently results in reduced levels of bacterial adsorption onto the hydrogel surfaces.

## 4. Conclusions

Herein, we demonstrate that the surface-functionalization of the p(HEMA) substrate with hydrophilic and zwitterionic MPC moieties is a viable approach for developing functional biomaterials with improved resistance to protein and bacterial adsorption. The hydrogels were prepared via three-step synthetic pathway consisting of synthesis of the p(HEMA) substrate, adsorption of a photosensitizer onto the hydrogel surfaces, and UV-induced free radical polymerization of the MPC monomers. The decrement in water contact angles of the MPC-modified hydrogels confirms the intrinsic ionic functionality of the incorporated p(MPC), which enhances the surface wettability of the prepared hydrogels. Remarkably, the presence of zwitterionic MPC moieties significantly decreases protein and bacterial adsorption to the modified hydrogels, due to the dense hydration layers formed by the polymerized MPC on the surfaces of the hydrogels. The results described herein demonstrate the feasibility of developing hydrogels that possess superior antifouling and antimicrobial properties. These novel multifunctional biomaterials demonstrate promising potential for various biomedical and biotechnological applications.

## Figures and Tables

**Table 1:** Characteristics of the poly(2-methacryloyloxyethyl phosphorylcholine)-surface-functionalized hydrogels.

Hydrogels	Amounts of Conjugated poly(MPC) (μmol/cm^2^ per Hydrogel Surface) ^1^	Equilibrium Swelling Ratio (%)^1^	Contact Angle (°) ^2^
p(HEMA) ^3^	–	60.1 ± 1.1	78.6 ± 9.6
p(HEMA)/p(MPC)0.1	18.0 ± 1.2	61.0 ± 2.9	74.7 ± 9.7
p(HEMA)/p(MPC)0.25	21.8 ± 0.5	61.6 ± 1.0	65.0 ± 4.7 ^4^
p(HEMA)/p(MPC)0.5	24.1 ± 1.8	68.3 ± 1.3	59.2 ± 7.0 ^4,5^

^1^ Data are means ± SD (n = 3). ^2^ Data are means ± SD (n = 10). ^3^ p(HEMA) is pristine p(HEMA)-based hydrogel. ^4^ p < 0.05 compared to p(HEMA). ^5^ p < 0.05 compared to p(HEMA)/p(MPC)0.1.
